# Le médicament générique au Maroc: le point de vue du consommateur

**DOI:** 10.11604/pamj.2013.15.18.2243

**Published:** 2013-05-09

**Authors:** Sanaa Zaoui, Farid Hakkou, Houda Filali, Youssef Khabal, Illias Tazi, Lahoucine Mahmal

**Affiliations:** 1 Laboratoire de Pharmacologie et de Toxicologie, Faculté de Médecine et de Pharmacie, Université Cadi Ayyad, Marrakech, Maroc; 2 Laboratoire de Recherche PCIM, Faculté de Médecine et de Pharmacie, Université Cadi Ayyad, Marrakech, Maroc; 3 Laboratoire de Pharmacologie et de Toxicologie, Faculté de Médecine et de Pharmacie, Casablanca, Maroc; 4 Faculté de Médecine et de Pharmacie de Fès, Maroc; 5 Service d'Hématologie, Centre Hospitalier-Universitaire Mohammed VI, Université Cadi Ayyad, Marrakech, Maroc

**Keywords:** Médicament générique, consommateur, perception, Maroc, generic drug, consumer, perception, Morocco

## Abstract

**Introduction:**

Le médicament générique est souvent une origine de préjugés négatifs et de méfiance chez les professionnels de santé et les patients. Ceci pourrait être dû à plusieurs facteurs entre autre le manque des connaissances du patient sur ces médicaments. Le but de notre étude était d’évaluer l'information du consommateur sur les médicaments génériques et apprécier leur utilisation de ces médicaments.

**Méthodes:**

Il s'agit d'une étude transversale réalisée de Janvier à Mars 2010 auprès de 251 sujets. Un questionnaire comprenant dix questions fermées a été utilisé. Les questions concernaient l’évaluation des connaissances sur les médicaments génériques, les sources d'information, le degré de confiance et d'information des patients sur ces médicaments. Une analyse descriptive simple des différentes variables a été réalisée.

**Résultats:**

Dans notre étude, 126 sujets (50,2%) ont répondu connaître les médicaments génériques. Parmi eux, 52,3% les utilisaient et 67,4% estimaient qu'ils étaient insuffisamment informés sur ces médicaments. Les cadres supérieurs et les étudiants ont représenté la catégorie qui utilisait le plus les médicaments génériques (respectivement dans 40,9% et 25,7%) et qui était la mieux informée (respectivement dans 61,9% et 23,81%). Le faible coût a été la principale motivation d'utilisation du médicament générique (93,9%). Les médias ont représenté la première source d'information (59,5%). Après sensibilisation, 88,8% des sujets qui ne connaissaient pas le médicament générique ont été favorable à son utilisation.

**Conclusion:**

Une promotion efficace par une politique d'information soutenue auprès des consommateurs et par des mesures incitatrices à la prescription et à la délivrance du générique par les médecins et les pharmaciens, pourront améliorer l'utilisation de ces médicaments dans notre pays.

## Introduction

Un médicament générique est la copie d'un médicament original qui a la même composition qualitative et quantitative en principe actif et la même forme pharmaceutique que la spécialité de référence [1]. Les médicaments génériques représentent 27% du marché pharmaceutique marocain, soit 68,5 millions d'unités vendues et près de 2 milliards de dirhams. Le gouvernement se fixe l'objectif d'un taux de pénétration du générique de 60% en 2015 dans le but de rationaliser les frais de dépenses en matière du médicament [2].

Seulement 30% de la population est couverte par une assurance maladie et le secteur public ne permet pas de pallier au déficit d'accessibilité au médicament [3]. De ce fait le développement du médicament générique est d'un intérêt capital, il permettra une économie du coût du traitement médical [4], sans qu'il y ait de préjudice pour le patient et revêt d'autres intérêts, entre autre le fait qu'il est parmi les meilleurs outils qui permettront la viabilité et la réussite de la couverture médicale de base dans notre pays [5].

Le médicament générique est souvent une origine de préjugés négatifs et de méfiance, de la part des professionnels de santé et des patients. Ils sont souvent considérés comme des médicaments de qualité inférieure, ceci pourrait être dû à plusieurs facteurs entre autre le manque des connaissances du patient sur ces médicaments [6]. Par conséquent, les consommateurs, leur connaissance sur les médicaments génériques et leur volonté de choisir les médicaments génériques sont actuellement d′importants déterminants de la réussite de développement du médicament générique [7].

L'influence du patient est un facteur déterminant dans la décision de prescription du médecin et dans la substitution par le pharmacien d'une molécule de référence par un générique [6]. L′utilisation de ces médicaments ne peut pas être améliorée sans la participation d'un consommateur informé. En plus de la fidélité à la marque, l′incompréhension en ce qui concerne le générique pourrait amener les consommateurs à préférer les médicaments de marque [6]. Il nous a semblé donc intéressant d’évaluer les connaissances du consommateur sur les médicaments génériques et d'apprécier leur utilisation de ces médicaments.

## Méthodes

Il s'agit d'une étude prospective réalisée sur une période de deux mois et demie de Janvier à Mars 2010 par un enquêteur auprès de 251sujets. Les questions ont été posées directement aux sujets par un seul et même enquêteur. On a utilisé un questionnaire comprenant dix questions. L’étude a été réalisée auprès des patients qui étaient hospitalisés ou venant consulter, ces patients étaient choisis au hasard dans divers hôpitaux publics et polycliniques privées de Casablanca. Nous avons également interrogé les étudiants de la Faculté de Médecine et Pharmacie de Casablanca. Les questions posées concernaient surtout l’évaluation des connaissances des sujets de l'enquête sur les médicaments génériques, leurs sources et degré d'information, leur avis vis-à-vis du médicament générique et leur utilisation de ces médicaments. Le questionnaire contenait principalement des questions fermées. Pour les patients qui ne connaissaient pas les médicaments génériques, nous leur avons donné la définition et nous leur avons demandé leur avis sur ces médicaments. Nous avons procédé à l'exploitation des données des questionnaires sur le logiciel Excel (Microsoft corporation, USA, version 2003).

## Résultats

### Données sociodémographiques

La majorité des patients (60,96%) étaient âgés de 25 à 50 ans, les plus de 50 ans ne représentaient que 14,74%. Les femmes dans cette étude représentaient 54,98%. La majorité des patients provenait du secteur public (53%). Les inactifs et les employés représentaient la tranche la plus importante, respectivement 27,09% et 26,69%. Les caractéristiques des sujets participant à l’étude sont présentées dans le Tableau 1.

**Tableau 1:** Caractéristiques des sujets qui ont participé à l’étude (n = 251)

Caractéristiques	N°	%
**Age**		
Moins de 25 ans	61	24,30
De 25 ans à 50 ans	153	60,96
Plus de 50 ans	37	14,74
**Sexe**		
Hommes	113	45,02
Femmes	138	54,98
**Catégories Socioprofessionnelles**		
Agriculteur	1	0,40
Commerçant	18	7,17
Cadre supérieur	33	13,15
Employés	67	26,69
Etudiant	52	20,72
Retraité	12	4,78
Inactif	68	27,09
**Lieu**		
Secteur public	133	53
FMPC	14	5,58
Secteur privé	104	41,42

### Connaissance des médicaments génériques par les sujets

Presque la moitié (50,20%) des sujets de l’étude estimaient connaître les médicaments génériques. Les caractéristiques des sujets qui connaissaient le médicament générique sont présentées dans le Tableau 2.

**Tableau 2:** Caractéristiques des sujets qui connaissaient le médicament générique (n= 126)

	N°	%
**Age**		
Moins de 25 ans	33	54,09
De 25 ans à 50 ans	84	54,90
Plus de 50 ans	9	24,32
**Sexe**		
Femmes	60	47,62
Hommes	66	52,38
**Catégorie socioprofessionnelle**		
Agriculteur	0	0,00
Commerçant	4	22,22
Cadre supérieur	32	96,96
Employé	32	47,76
Etudiant	37	71,15
Retraité	4	33,33
Inactif	17	25,00

### Utilisation des médicaments génériques

Sur les 126 sujets qui connaissaient les médicaments génériques, 66 (52,38%) les utilisaient. Les caractéristiques des sujets qui utilisaient les médicaments génériques sont présentées dans le Tableau 3. La plupart des sujets (63,64%) qui utilisaient les médicaments génériques faisaient eux-mêmes la demande pour se procurer ces médicaments. Dans 31,82% des cas les médecins prescrivaient les médicaments génériques aux sujets, alors que dans 9,09% des cas seulement les pharmaciens les leurs conseillaient. Les médicaments génériques ont été utilisés principalement en raison de leur coût qui est bas (93,94%). Dans 34,85% des cas les sujets de l’étude trouvaient ces médicaments efficaces, seulement 10,61% trouvaient que ces médicaments sont bien tolérés. Environ 48% des sujets n'utilisaient pas les génériques et ceci pour diverses raisons: ils sont différents de leurs médicaments (38,33%), ils ne font pas confiance à ces médicaments (26,67%), ils sont inefficaces (16,67%).

**Tableau 3:** Répartition des sujets utilisant le médicament générique (n = 66)

	N°	%
**Age**		
Moins de 25 ans	16	24,24
De 25 ans à 50 ans	45	68,18
Plus de 50 ans	5	7,58
**Sexe**		
Femmes	24	36,36
Hommes	42	63,64
**Catégorie socio-professionnelle**		
Agriculteur	0	0,00
Cadre supérieur	27	40,91
Commerçant	2	3,03
Employé	13	19,70
Etudiant	17	25,76
Retraité	2	3,03
Inactif	5	7,58

### Information sur les médicaments génériques

Sur les 126 sujets connaissant les médicaments génériques, 73 (57,94%) ont déclaré avoir déjà vu un support publicitaire sur ces médicaments. Les sujets ont été réparti entre ceux qui estimaient ne pas être du tout informés, insuffisamment informés et suffisamment informés sur les médicaments génériques. Le média le plus souvent cité était la télévision. Les sujets de l’étude avaient besoin d'avoir des informations sur l'efficacité, l’économie réalisé par le médicament générique et ses effets indésirables (Tableau 4). Parmi les sujets de l’étude 21 estimaient qu'ils sont tout à fait informés sur les médicaments génériques. Les hommes (71,43%), les patients âgés de 25 à 50 ans (71,43%) et les cadres supérieurs (61,90%) sont les mieux informés. Pour les patients qui ne connaissaient pas les médicaments génériques nous leur en avons donné la définition et nous avons demandé leur avis sur ces médicaments. La plupart des patients étaient intéressés et favorables à l'utilisation de ces médicaments après leur avoir expliquer la définition d'un médicament générique et ceci dans 88,8% des cas.

**Tableau 4:** Répartition des patients en fonction de leur degré et source d'information et le type d'information souhaité (n = 126)

	Effectif	Pourcentage
**Degré d'information**		
Pas du tout	20	15,87
Insuffisamment	85	67,46
Tout à fait	21	16,67
**Source d'information**		
Médecin	16	12,70
Pharmacien	14	11,11
Famille	10	7,94
Sec. Sociale	2	1,59
Médias : TV	75	59,52
Délégués Médicaux	6	4,76
Enseignants	31	24,60
**Type d'information souhaitée**		
Composition	25	19,84
Economie	45	35,71
Effets secondaires	44	34,92
Efficacité	101	80,16
Autres	3	2,38

## Discussion

Presque la moitié des participants à l′enquête n′étaient pas familiers avec le terme générique. Bien que ce taux est encore faible il s'est bien amélioré si on fait une comparaison avec une étude réalisée sur la perception des génériques par la population marocaine au niveau de la région de Casablanca entre Mars et Juin 2005 auprès d'un échantillon de 150 patients, ou on a trouvé que 85% des patients interrogés n'ont jamais entendu parler d'un médicament générique [8]. Cette amélioration s'explique par le fait que ces dernières années, le ministère de la santé en collaboration avec l'Agence nationale de l'assurance maladie ont organisé une campagne de sensibilisation et d'information à l'utilisation des médicaments génériques ainsi que la promotion des génériques par le biais de séminaires de formation et de spots publicitaires.

Nos résultats rejoignent ceux d'une enquête faite à Auckland en 2008 et qui a trouvé que 51.6% des sujets de l'enquête connaissait les médicaments génériques [6]. Dans une autre étude 68,4% des sujets de l'enquête connaissaient le terme « médicaments génériques» [9]. Une enquête en Malaisie a montré qu'il y avait des lacunes dans les connaissances des consommateurs en ce qui concerne les médicaments génériques [10].

Les inactifs qui sont les plus susceptibles d′avoir un faible revenu et qui devaient être donc les consommateurs les plus importants de ces produits étaient moins familiers avec les génériques, seulement 13% de cette catégorie connait ce médicament.

Les cadres supérieurs, les étudiants et les employés connaissaient mieux les médicaments génériques. Des résultats similaires en été trouvé dans l’étude faite à Auckland ou les patients les plus jeune, actifs et ayant un niveau d’éducation élevé sont les plus informés sur les génériques [6].

La source d'information la plus citée était les médias; le plus souvent la télévision. L’état a depuis quelques années mené des compagnes publicitaires sur le médicament générique. Parmi les sources d'information, les médecins et les pharmaciens étaient respectivement en 3ème et 4ème position. On remarque que ces derniers n'informent pas assez leurs patients. Des résultats proches des nôtres concernant la communication des médecins et des pharmaciens sur les médicaments génériques ont été trouvés dans une étude ou seulement 19,6% des répondants ont répondu que leurs médecins leur parlent des médicaments génériques et 24,2% ont indiqué que leurs pharmaciens s′engagent dans de telles discussions [11].

A la différence de notre étude, d'autres études ont trouvé que les pharmaciens étaient la première source d'information des consommateurs du fait de leurs connaissances sur ces médicaments et leur contact fréquent avec les patients. Les médecins, se trouvent en deuxième ligne pour informer les consommateurs [6]. Grace aux mesures prise par le gouvernement en Australie, les pharmaciens recommandent les génériques pour 96,4% des produits d′ordonnance qui sont éligibles pour la substitution [12].

Les médecins et les pharmaciens ont un rôle clef à jouer qui est de bien informer leurs patients sur l'intérêt de l'utilisation des médicaments génériques mais il faut qu'ils soient eux aussi motivés par les génériques, c'est pour cela qu'il faut intensifier l'information vers cette cible. Si les décideurs espèrent augmenter l'utilisation des médicaments génériques, une intervention auprès des praticiens est nécessaire. Une formation continue, une meilleure communication avec les prescripteurs et les pharmaciens et des campagnes d′éducation peuvent être plus efficaces si elles se concentrent sur ces 2 partenaires et pourrait les motiver à prescrire et à délivrer le générique [13]. Une enquête faite auprès des médecins marocains en 2006 a trouvé qu'une bonne partie de ces médecins n’était pas bien informée sur les médicaments génériques [5].

Une bonne partie des sujets qui connaissaient les médicaments génériques estiment qu'ils sont insuffisamment informés sur ces médicaments (67%). Ces sujets ont indiqué qu′ils voulaient être plus informés sur ce sujet (efficacité 80%, économies 36%). Nos résultats rejoignent ceux d'une autre étude qui a montré que 94% des sujets de l'enquête ont déclaré avoir besoin d′être mieux informés sur les médicaments génériques [6]. Après information la majorité des participants à notre enquête (88,80%) étaient prêts à utiliser un médicament générique.

La littérature rapporte que des liens ont été établi entre les connaissances du patient au sujet des génériques avec l′utilisation réelle de ces médicaments, de ce fait les décideurs devraient prendre conscience des avantages des campagnes d′éducation qui peuvent influencer les perceptions du consommateur envers le médicament générique [14].

Quant à la sécurité sociale, elle n’était pratiquement jamais citée par les patients interrogés alors qu'elle a un rôle primordial d'information des patients sur les médicaments génériques. Ceux qui utilisaient le plus les génériques sont les hommes, les âgés de 25 à 50 ans et les cadres supérieurs. Ils représentent la catégorie des sujets qui a déclaré le plus qu'elle était tout à fait informée sur les génériques.

Parmi les patients utilisant les génériques seulement 34% des patients estimaient qu'ils étaient efficaces et peu de patients, environ 10% trouvaient ces médicaments bien tolérés. La méfiance concernant l'efficacité du générique a été retrouvé également dans d'autres études [15, 16]. Dans d'autres études et à la différence de la nôtre le médicament générique n’était considéré moins efficace que chez 26,4% des sujets [17]. La motivation principale de l'utilisation était le moindre cout qui a été cité par la majorité des sujets, nos résultats rejoignent ceux trouvés par d'autres études [11].

Environ 48% des patients connaissant les génériques ne l'utilisaient pas et ceci pour diverses raisons: ils sont différents de leur médicaments (38,33%), ils ne font pas confiance à ces médicaments (26,67%), ils sont inefficaces (16,67%). La méfiance concernant la qualité du médicament générique à également été retrouvé dans une autre étude ou 28% des personnes interrogées pensent qu'il existe une différence de qualité entre le médicament et son générique [15].

Des études ont montré que l′attitude des consommateurs vers les médicaments génériques est largement déterminée par leur compréhension du terme générique et la confiance que les médicaments génériques ont la même qualité, efficacité et sécurité [18].

## Conclusion

En vue d'atteindre l'objectif de rationaliser les frais sanitaires à travers l'implantation des médicaments génériques au Maroc, plusieurs mesures sont à prendre. D'abord il faut rassurer les patients, quant à la qualité pharmaceutique et l’équivalence des médicaments génériques et leur expliquer le rôle du générique dans la régulation des dépenses de santé et son impact économique. Les médecins et les pharmaciens sont les principaux piliers et sources d'information pour les patients, il faut intensifier l'information destinée à ces deux partenaires et mettre en place des mesures permettant d'encourager ces derniers, à prescrire et à dispenser les médicaments génériques, en particulier le droit de substitution.

## References

[1] Lagarce L (2005). Médicaments génériques, le point de vue des médecins: enquête d'opinion réalisée auprès des médecins libéraux du Maine-et-Loire. Thérapie..

[2] Abashi Shamamba Médicaments génériques. Comment reconquérir le corps médical. L’économiste, édition N 3479 du 2011/03/04. http://www.leconomiste.com/article/medicaments-generiquesbrcomment-reconquerir-le-corps-medical?page=3.

[3] Khayati Y Médicaments génériques leurs problématiques. http://pharmacies.ma/pharmacie/upload/Sections/file/khiyati.pdf.

[4] Berg MJ, Gross RA, Haskins LS, Zingaro WM (2008). Generic substitution in the treatment of epilepsy: Patient and physician perceptions. Epilepsy Behav..

[5] Zaoui S, Hakkou F, et Filali H (2011). Le médicament générique au Maroc: enquête auprès des médecins. Thérapie..

[6] Babar ZU, Stewart J, Reddy S, Alzaher W (2010). An evaluation of consumers’ knowledge, perceptions and attitudes regarding generic medicines in Auckland. Pharm World Sci..

[7] Sharrad AK, Hassali MA (2011). Consumer perception on generic medicines in Basrah, Iraq: Preliminary findings from a qualitative study. Res Social Adm Pharm..

[8] La perception des génériques par la population http://www.repere-medical.com/article-107.html.

[9] Kobayashi E, Karigome H, Sakurada T (2011). Patients’ attitudes towards generic drug substitution in Japan. Health Policy..

[10] Al-Gedadi NA, Hassali MA, Shafie AA (2008). A pilot survey on perceptions and knowledge of generic medicines among consumers in Penang, Malaysia. Pharmacy Practice..

[11] Shrank WH, Cox ER, Fischer MA, Mehta J (2009). Patients’ Perceptions of Generic Medications. Health Aff (Millwood)..

[12] Chong CP, March G, Clark A, Gilbert A (2011). A nationwide study on generic medicines substitution practices of Australian community pharmacists and patient acceptance. Health Policy..

[13] Vallès JA, Barreiro M, Cereza G, Ferro JJ (2003). A prospective multicenter study of the effect of patient education on acceptability of generic prescribing in general practice. Health Policy..

[14] Shrank WH, Cadarette SM, Cox E, Fischer MA (2009). Is There a Relationship Between Patient Beliefs or Communication About Generic Drugs and Medication Utilization?. Med Care..

[15] Sarradon-Eck A, Blanc MA, Faure M (2007). Users sceptical about generic drugs: an anthropological approach. Rev Epidemiol Sante Publique..

[16] Iosifescu A, Halm EA, McGinn T, Siu AL (2008). Beliefs about generic drugs among elderly adults in hospital-based primary care practices. Patient Educ Couns..

[17] Fabiano V, Mameli C, Cattaneo D, Delle Fave A (2012). Perceptions and patterns of use of generic drugs among Italian Family Pediatricians: First round results of a web survey. Health Policy..

[18] Palagyi M, Lassanova M (2008). Patients attitudes towards experience with use of generics in Slovakia, performance of generic substitution. Bratisl Lek Listy..

